# Reduced Honeybee Pollen Foraging under Neonicotinoid Exposure: Exploring Reproducible Individual and Colony Level Effects in the Field Using AI and Simulation

**DOI:** 10.1021/acs.est.4c13656

**Published:** 2025-03-07

**Authors:** Ming Wang, Frederic Tausch, Katharina Schmidt, Matthias Diehl, Silvio Knaebe, Holger Bargen, Farnaz Faramarzi, Volker Grimm

**Affiliations:** † Department of Ecological Modelling, Helmholtz Centre for Environmental Research—UFZ, Permoserstr. 15, 04318 Leipzig, Germany; ‡ apic.ai GmbH, Melanchthonstraße 2, 76131 Karlsruhe, Germany; § FZI Research Center for Information Technology, Haid-und-Neu-Str. 10-14, 76131 Karlsruhe, Germany; ∥ Eurofins Agroscience Services Ecotox GmbH, Eutinger Street 24, 75223 Niefern-Öschelbronn, Germany; ⊥ Department of Plant Ecology and Nature Conservation, University of Potsdam, Zeppelinstraße 48 A, 14471 Potsdam-Golm, Germany

**Keywords:** Apis mellifera, automated monitoring, computational modeling, feeding study design, neurotoxic effect, Oomen study, pesticide exposure, pollen foraging

## Abstract

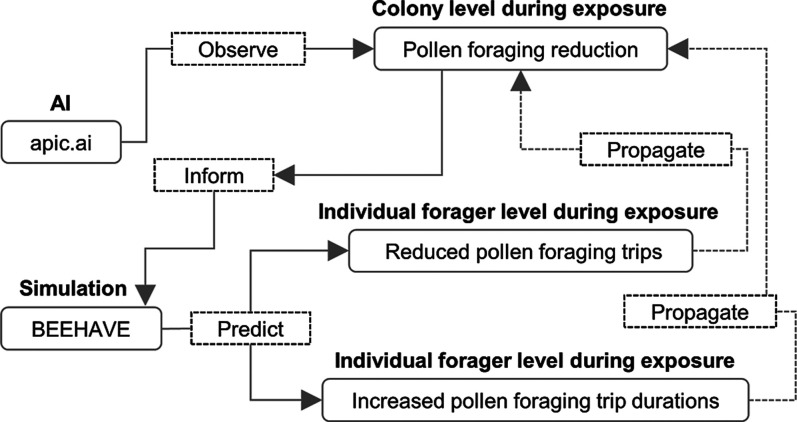

Honeybees (*Apis mellifera*) are important pollinators. Their foraging behaviors are essential to colony sustainability. Sublethal exposure to pesticides such as neonicotinoids can significantly disrupt these behaviors, in particular pollen foraging. We investigated the effects of sublethal doses of the neonicotinoid imidacloprid on honeybee foraging, at both individual and colony levels, by integrating field experiments with artificial intelligence (AI)-based monitoring technology and mechanistic simulations using the BEEHAVE model. Our results replicated previous findings, which showed that imidacloprid selectively reduces pollen foraging at the colony level, with minimal impact on nectar foraging. Individually marked exposed honeybees exhibited prolonged pollen foraging trips, reduced pollen foraging frequency, and instances of drifting pollen foraging trips, likely due to impaired cognitive functions and altered metabolism. These behavioral changes at the individual level corroborated the previous model predictions derived from BEEHAVE, which highlights the value of combining experimental and simulation approaches to disentangle underlying mechanisms through which sublethal effects on individual foragers scale up to impact colony dynamics. Our findings have implications for future pesticide risk assessment, as we provide a robust feeding study design for evaluating pesticide effects on honeybee colonies and foraging in real landscapes, which could improve the realism of higher-tier ecological risk assessment.

## Abstract 2

Honeybees typically collect either nectar or pollen during foraging trips. This study shows neonicotinoid insecticides specifically impair pollen foragers’ performance within a colony, highlighting important implications for pesticide risk assessments.

## 1 Introduction

Honeybees (*Apis mellifera*) are essential pollinators that play a crucial economic and ecological role in global agriculture and natural ecosystems.^1−3^ However, honeybee colony health has been threatened by multiple stressors and their interactions, including climate change, habitat loss, parasites and pathogens, pesticide exposure, and poor management practices.^2,4−6^ Most of these stressors directly or indirectly affect honeybee foraging, which strongly affects the survival of a colony. Foraging worker bees collect nectar providing carbohydrates for energy and pollen serving as a source of proteins and other nutrients, particularly for the brood and the queen bee.^7−9^ This foraging is vital for maintaining the colony’s health by ensuring a steady supply of food and nutrients necessary for growth and reproduction. Without effective foraging, the colony would struggle to sustain itself and eventually collapse.

There is strong evidence that highly potent neuroactive pesticides, such as neonicotinoids, can have sublethal effects on honeybee foraging behaviors, even when bees are exposed to low, field-relevant concentrations.^10^ For example, foragers exposed to these chemicals may take longer to complete their foraging trips,^11,12^ which means they would spend more time searching for resources and navigating the environment. As a result, exposed bees may make fewer foraging trips^13−15^ throughout the day, which would reduce the total amount of resources brought back to the colony. In addition, Prado et al.^16^ showed that a mixture of pesticides, including fungicides and insecticides, reduced the amount of pollen collected by individual bees, but did not affect the amount and concentration of nectar gathered. Furthermore, some foragers exposed to pesticides experience difficulties with homing, a critical cognitive function that allows bees to locate and return to their hive.^17−19^ This loss further reduces the colony’s workforce.

These changes in foraging behaviors, therefore, suggest that pesticide exposure impacts not only bees’ navigational abilities but also their efficiency in resource collection, in particular for pollen. This effect may occur through interference with cognitive processes and metabolic functions, which are essential mechanisms on which bees rely for decision-making and sustained flight. Another possible indication of cognitive impairment induced by pesticide exposure is the phenomenon known as “drifting”, where foragers inadvertently return to colonies other than their own.

However, most of the existing studies investigating the sublethal effects of these pesticides have focused on individual foragers, using mark-and-track and catch-and-release approaches (e.g., Ohlinger et al.,^20^ Shi et al.,^21^ and Henry et al.^22^). Although these studies have identified significant changes in individual behaviors and provide valuable insights into how pesticides affect foraging time, navigational accuracy, and foraging efficiency, they have not yet established a clear link between these individual-level effects and the overall foraging performance at the colony level. This missing link highlights a critical research gap in our understanding: How do the sublethal effects observed at the individual foragers translate into measurable impacts on the colony’s ability to forage as a collective unit?

To address this, we conducted a field study in 2019 (referred to as the 2019 feeding experiments) to investigate the effects of pesticide exposure on colony-wide foraging behaviors,^23^ using our newly developed camera and AI-based monitoring system, i.e., the apic.ai monitoring technology^24^ that enables us to count foragers leaving and entering a hive and to distinguish between those with and without pollen pellets. We were able to track and quantify the foraging activities of the entire colony under sublethal doses of the neonicotinoid imidacloprid. Our field study provided the first evidence that field-realistic doses of imidacloprid specifically reduced pollen foraging of the entire colony while having little to no effect on nectar foraging.^23^ To understand the mechanisms underlying this finding, we then used the mechanistic simulation model—BEEHAVE.^25^ The simulations replicated the observed foraging activities surprisingly well, when we assumed that imidacloprid increased the foraging trip durations of pollen foragers.^23^

In the current study, we replicated the 2019 feeding experiments in 2023 (referred to as the 2023 feeding experiments) to investigate whether the previously observed reduction in pollen foraging at the colony level can be consistently reproduced. We also aimed to test model predictions derived from the BEEHAVE simulations by examining the effects of imidacloprid at the individual level (referred to as the 2023 Reidentification experiments). The 2023 Reidentification experiments involved marking individual bees and tracking their foraging trips, trip durations, and occurrences of drifting, as well as recruitment of newly emerged bees, using the apic.ai monitoring technology.^24^ Our goals are thus (1) to test whether the design of our feeding experiments provides reproducible results, which, given the notoriously high variability in behaviors and traits within honeybee colonies,^26^ would be an important finding, as it could serve as a basis for tests supporting regulatory risk assessment of pesticides and (2) to test the model predictions, thereby gaining insights into the effects of pesticides on pollen foraging at both the colony and individual levels.

## 2 Materials and Methods

### 2.1 2023 Feeding Experiments

The 2023 feeding experiments were conducted between 21 July 2023 and 25 August 2023. To replicate the 2019 feeding experiments,^23^ eight Zander colonies of free-flying honeybees in an agricultural landscape near Bretten, southwestern Germany (48.916780°N, 8.713613°E), were provided with 500 g of 50% sugar solution daily from a feeder within the hives. The feeding lasted for 11 consecutive days, from 28 July 2023 to 07 August 2023. Each colony had two bodies with 10 frames containing all brood stages (i.e., eggs, larvae, and capped cells) with the brood nest confined to one body. The queen bees were 2 years old. The hives were placed in pairs on wooden pallets at the same site and faced identical environmental conditions (Figure S1), and each hive was equipped with the apic.ai monitoring technology.^24^

Four of the eight colonies served as the Control group (Control), while the remaining four were assigned to the Treatment group (Treatment). In Treatment, nonformulated imidacloprid (Molecular formula: C_9_H_10_ClN_5_O_2_, CAS number: 138261-41-3, Thermo Fisher Scientific Inc.) was used at the same concentration as in the 2019 experiments (200 μg imidacloprid/kg sugar solution − 200 ppb),^23^ which is known to be a sublethal dose.^27^ The Control colonies were fed only with 500 g of 50% sugar solution, without imidacloprid. At the start of the study on 25 July 2023, both Control and Treatment hives had similar overall colony conditions, including colony strength (i.e., the number of adult worker bees) (Figure S2), brood amount (Figure S3), and food resource availability in hives (Figures S4 and S5).

This study design ensured that any statistical differences observed between the Control and Treatment were due to the neonicotinoid imidacloprid rather than to other external or internal factors affecting the hives. Throughout the preexposure, exposure, and postexposure periods, daily colony-level foraging activities were monitored using the apic.ai monitoring technology,^24^ specifically the number of returning pollen foragers and nonpollen foragers (i.e., nectar and water foragers, scouts, guards, etc. which can mostly be assumed to have been nectar foragers). The number of these returning foragers was the focus for rigorous statistical analysis because there were no significant differences in overall colony conditions, including the number of adult worker bees (Figure S2), between Control and Treatment before imidacloprid exposure. In addition, adult worker bee mortality was assessed daily for each hive by counting the number of dead adult workers in both the dead bee trap and the bottom drawer. According to Imdorf et al.,^28^ colony assessments, including counts of adult workers, brood cells, nectar cells, and pollen cells, as well as evaluations of sugar solution consumption, were conducted on five dates during the study: 25 July 2023; 04 August 2023; 11 August 2023; 17 August 2023; and 24 August 2023. The weather data during the study period are shown in Appendix B in Supporting Information.

### 2.2 BEEHAVE Simulations

BEEHAVE^25^ is a computer model, which was designed to explore various stressors affecting honeybee colonies, both individually and in combination. It links in-hive dynamics with land use in surrounding landscapes and weather conditions through honeybee foraging. In our previous BEEHAVE simulations,^23^ the model successfully reproduced colony-level pollen foraging dynamics observed over the study period, when the parameter (TIME_POLLEN_GATHERING) was varied from 600 to 6000 s during the exposure period. This parameter represents the time a pollen forager spends in a pollen-abundant flower patch to collect a pollen load.^25^

The model predictions derived from our BEEHAVE simulations^23^ can be interpreted as follows. An increase in the parameter (TIME_POLLEN_GATHERING) led to a longer overall pollen foraging trip duration for each forager. As a result, within a given foraging period (i.e., a set number of hours under favorable weather conditions), longer trip durations reduced the total number of foraging trips that a forager could complete in a day. This, in turn, decreased the number of outgoing foragers and returning foragers per day, which ultimately inhibited overall colony-level foraging activities.

These model predictions provided plausible individual-level mechanisms underlying the observed reduction in colony-level pollen foraging, which enabled us to test them in the 2023 Reidentification experiments (see Section 2.3).

### 2.3 2023 Reidentification Experiments

In these experiments, we used colonies and settings from the 2023 feeding experiments. A total of 428 bees [203 foragers, and 225 newly emerged bees (48 h old or younger after hatching)] from two replicates (Ca and Cb) in Control and 436 bees [216 foragers, and 220 newly emerged bees (48 h old or younger after hatching)] from two replicates (Ta and Tb) in Treatment were marked with tags glued to their thorax on 26 − 27 July 2023 during the preexposure period and tracked using the apic.ai monitoring technology^24^ until the end of the study. This allowed us to assess whether there were any potential detrimental effects at the individual level that occurred, such as changes in trip durations, numbers of foraging trips, and recruitment of newly emerged bees as well as occurrences of drifting trips.

Each bee was marked with a unique identifier using custom-printed opalith plates, which were detectable by the apic.ai camera system.^24^ The system captured images of the marked bees as they entered and exited the hive. The reidentification process relied on an algorithm with a high detection accuracy. For each marked bee, individual-level observations were recorded, including trip durations, numbers of foraging trips, recruitment of newly emerged bees, and occurrences of drifting trips.

One limitation of the reidentification process was its inability to recognize bees passing through the camera’s field of view upside down, which could lead to underestimations of trip durations. However, the process ensured that whenever a marked bee was detected, it was certain that the bee was active, thereby reducing uncertainties about its activity level and survival. The details of the marking-and-tracking method are described in Appendix A in Supporting Information.

### 2.4 Data Analysis

The data collected on daily adult worker bee mortality, colony assessments (i.e., the number of adult worker bees, brood cells, nectar cells, and pollen cells, and the amount of sugar solution consumed), colony-level foraging activities (i.e., the number of returning pollen foragers and nonpollen foragers), and individual marked bees (i.e., the trip duration and the number of foraging trips) were first checked for normality using the Shapiro–Wilk test. Depending on the result, either the Mann–Whitney *U* test or the Welch’s *t*-test was applied to assess significant differences between Control and Treatment, as recommended by Ruxton.^29^ When the sample size was small (*n* = 4), the Welch’s *t*-test was used.

The drifting data were analyzed using the exact binomial test for observed counts to assess proportions between groups. This enabled us to determine whether any group had more drifting trips than another. The exact binomial test was also similarly used to assess differences in the mortality of individually marked bees between the two groups, which allowed us to determine if any group had a higher number of dead marked bees counted than another.

R^30^ and the packages “readxl”,^31^ “tidyverse”,^32^ “lubridate”,^33^ “ggstatsplot”,^34^ “ggpubr”,^35^ “rstatix”,^36^ “ggprism”,^37^ and “cowplot”^38^ were used to analyze and visualize the data mentioned above.

In addition, the Kaplan–Meier method (time-to-event curves) and the log-rank test were applied to visualize and analyze the data for the difference in the recruitment of newly emerged bees for pollen foraging between the two groups, using Python^39^ with the packages “lifelines”,^40^ “pandas”^41^, and “matplotlib”.^42^

## 3 Results

### 3.1 Effects at the Colony Level

#### 3.1.1 Colony-Level Foraging

**Figure 1:**
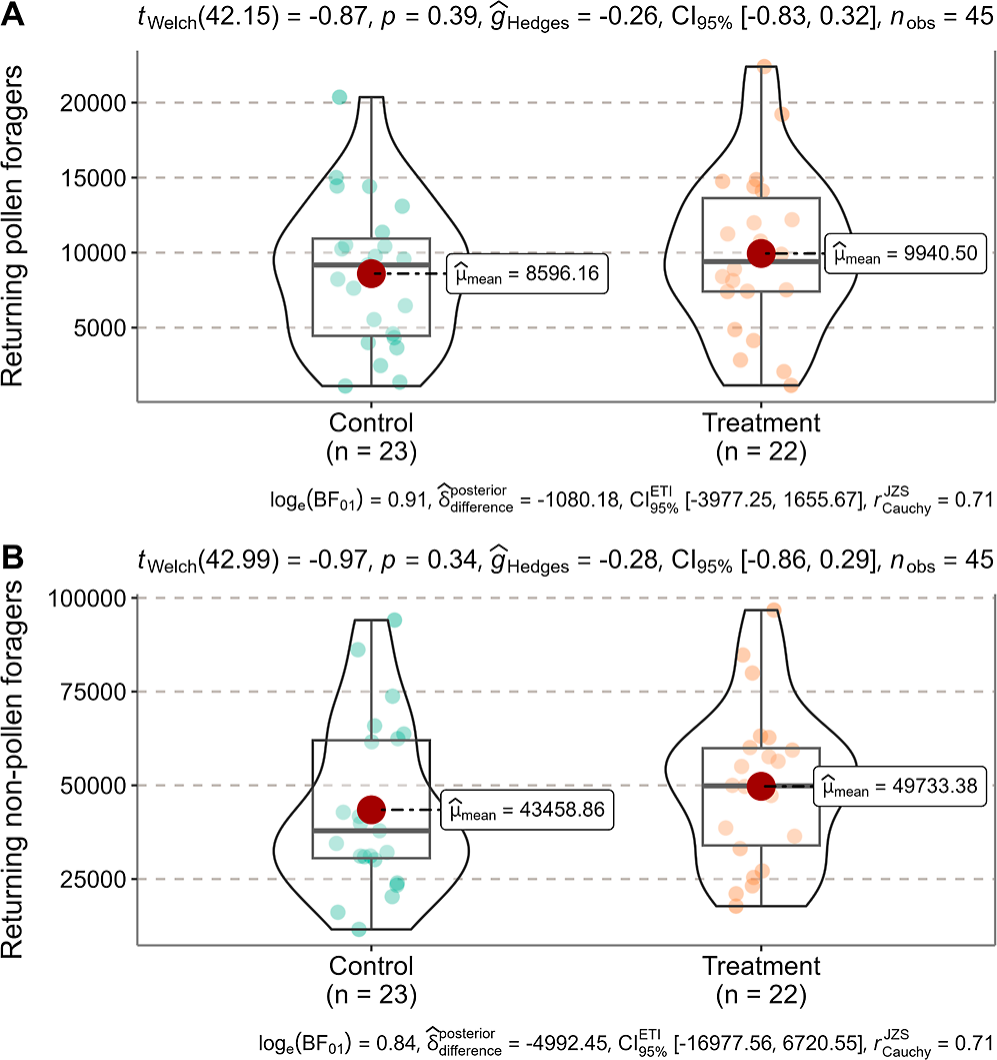
Comparison of the number of returning pollen foragers (A) and nonpollen foragers (B) between Control and Treatment during the preexposure period (21 − 27 July 2023) by the Welch’s *t*-test (not significantly different at *P* = 0.05). Each point represents the number of returning pollen or nonpollen foragers per hive per date in the Control or Treatment hives.

**Figure 2:**
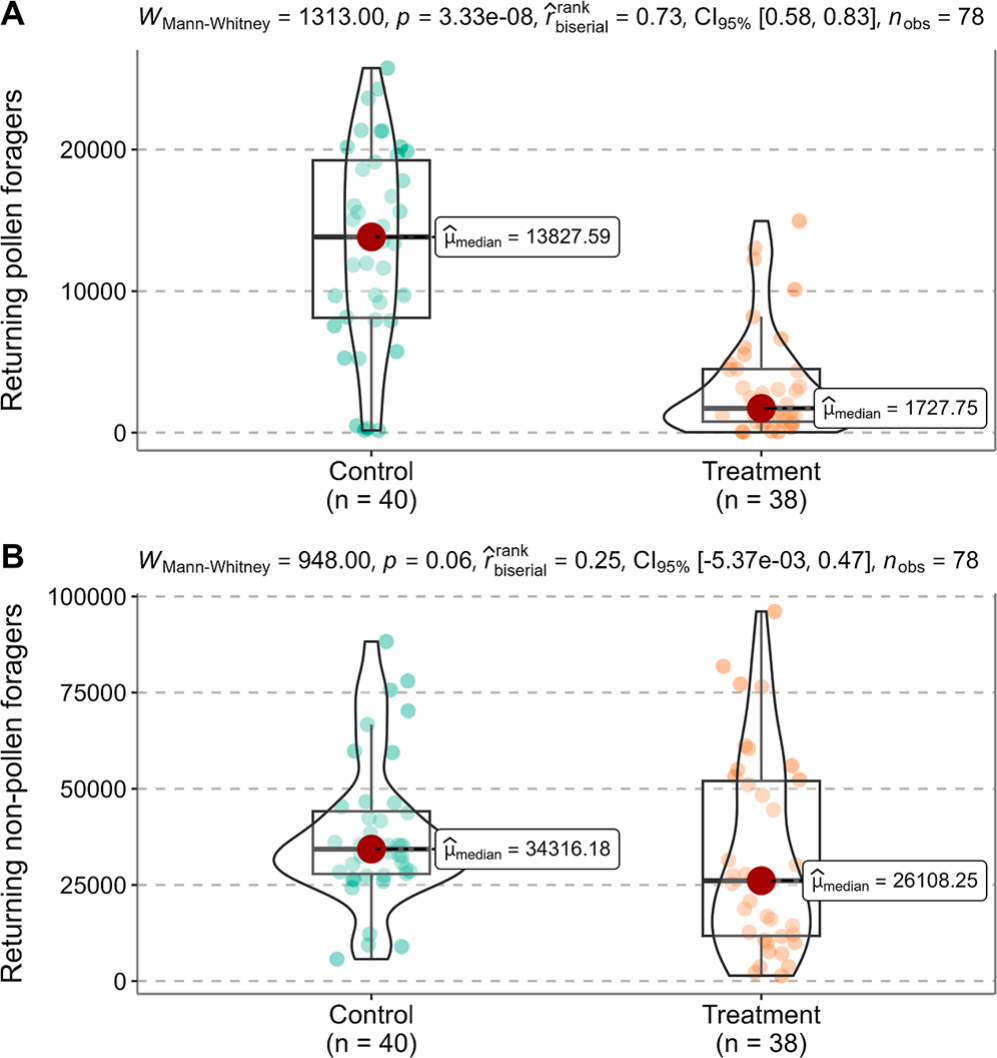
Comparison of the number of returning pollen foragers (A) and nonpollen foragers (B) between Control and Treatment during the exposure period (28 July 2023 − 07 August 2023) by the Mann–Whitney *U* test (not significantly different at *P* = 0.05). Each point represents the number of returning pollen or nonpollen foragers per hive per date in the Control or Treatment hives.

**Figure 3:**
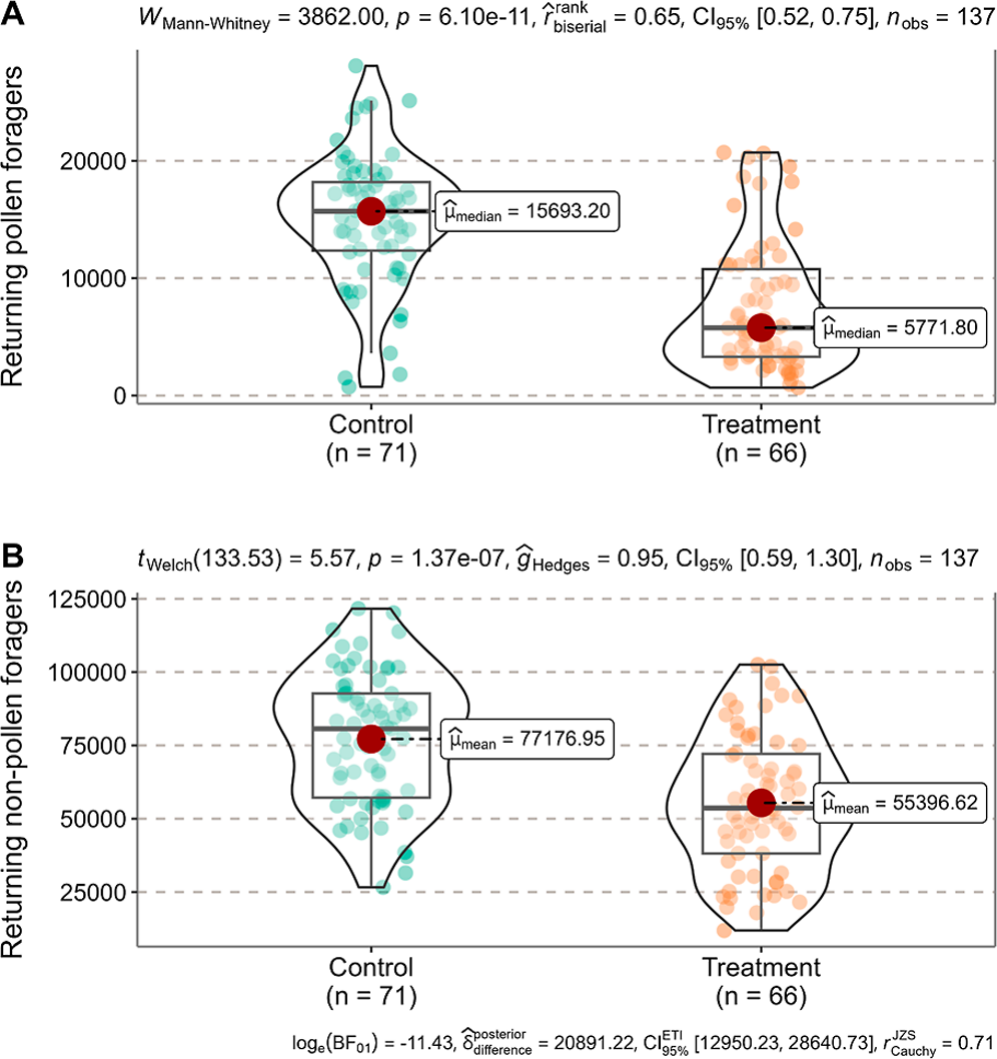
Comparison of the number of returning pollen foragers (A) and nonpollen foragers (B) between Control and Treatment during the postexposure period (08 − 25 August 2023) by the Mann–Whitney *U* test and the Welch’s *t*-test (not significantly different at *P* = 0.05). Each point represents the number of returning pollen or nonpollen foragers per hive per date in the Control or Treatment hives.

During the preexposure period, there was no significant difference in the number of returning pollen or nonpollen foragers between Control and Treatment (Figure 1). For both groups, returning pollen foragers accounted for approximately 20% of returning nonpollen foragers [mean ± SD (*n* = 23): 8596 ± 4899 out of 43,459 ± 22,241 in Control; mean ± SD (*n* = 22): 9941 ± 5397 out of 49,733 ± 21,005 in Treatment]. However, during the exposure period, the number of returning pollen foragers in Treatment was significantly lower than that in Control (Figure 2A). Meanwhile, the number of nonpollen foragers in Treatment did not differ from that in Control (Figure 2B). In Control, returning pollen foragers were approximately 40% of returning nonpollen foragers [median (IQR) (*n* = 40): 13,828 (8120 − 19,243) out of 34,316 (27,868 − 44,148)], whereas they made up only about 7% in Treatment [median (IQR) (*n* = 38): 1728 (774 − 4494) out of 26,108 (11,763 − 52,030)]. In the postexposure period, fewer returning pollen or nonpollen foragers were found in Treatment compared with Control (Figure 3). Returning pollen foragers comprised approximately 20% of returning nonpollen foragers in Control [mean ± SD (*n* = 71): 15,110 ± 5542 out of 77,177 ± 22,511] and about 11% in Treatment [median (IQR) (*n* = 66): 5772 (3296 − 10,762) out of 53,650 (38,171 − 72,091)].

#### 3.1.2 Adult Worker Mortality

We found no significant difference in the daily mortality of adult workers between Control and Treatment on most dates during the study period (21 July 2023 − 25 August 2023) (Table S1). However, on one date during the exposure period (01 August 2023), the number of dead adult workers in Treatment was significantly higher than that in Control (Table S1). This difference was due to a lower number of dead adult workers in Control on that date, while the mortality in Treatment on the same date remained within natural variability.

#### 3.1.3 Colony Assessment

Throughout the preexposure, exposure, and postexposure periods, there was no significant difference in colony strength (i.e., the number of adult worker bees) between Control and Treatment (Figure S2). However, during the postexposure period, the number of brood cells in Treatment was significantly reduced, compared with Control (Figure S3). Similarly, there was a significant decrease in the number of pollen cells in Treatment during this period compared to that in Control (Figure S4C). In addition, a considerably lower number of pollen cells tended to be found in Treatment during the exposure period compared with Control, although this difference was not statistically significant (Figure S4B). By contrast, the number of nectar cells in Treatment was significantly higher than that in Control during the postexposure period (Figure S5). As for sugar solution consumption, bees in Control consumed more feeding solution than those in Treatment (Figure S6).

### 3.2 Effects at the Individual Level

#### 3.2.1 Pollen Foraging Trip Durations

**Figure 4:**
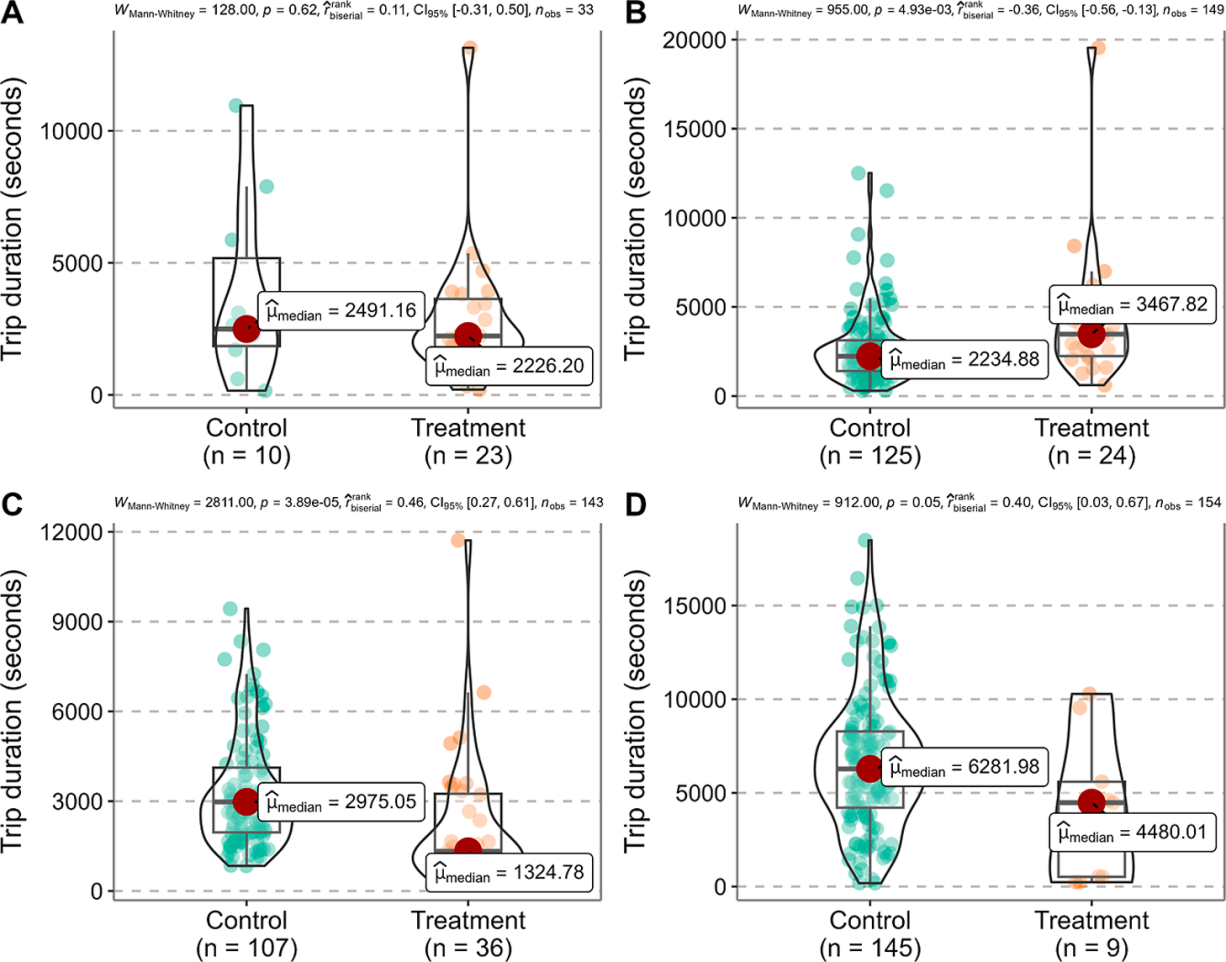
Comparison of the trip duration of marked foragers or newly emerged bees returning with pollen between Control and Treatment by the Mann–Whitney *U* test (not significantly different at *P* = 0.05). (A) Marked foragers during the preexposure period (21 − 27 July 2023). (B) Marked foragers during the exposure period (28 July 2023 − 07 August 2023). (C) Marked foragers during the postexposure period (08 − 25 August 2023). (D) Marked newly emerged bees during the postexposure period (08 − 25 August 2023). Each point refers to the trip duration of each marked forager or newly emerged bee returning with pollen per hive per date in the Control or Treatment hives.

The pollen foraging trip durations for marked foragers in both Control and Treatment were similar before the exposure (Figure 4A). However, during the exposure period, marked foragers in Treatment took significantly longer to complete their pollen foraging trips compared to those in Control (Figure 4B). After the exposure, marked foragers in Treatment spent less time on completing their pollen foraging trips than those in Control (Figure 4C). In contrast, during the postexposure period, there was no difference in the pollen foraging trip durations of marked newly emerged bees between Treatment and Control (Figure 4D). No observations were recorded for marked newly emerged bees engaged in pollen foraging in Treatment during the preexposure and exposure periods, while zero and three bees were recorded in Control during the preexposure and exposure periods, respectively. Therefore, statistical analysis could not be conducted.

#### 3.2.2 Pollen Foraging Trips

**Figure 5:**
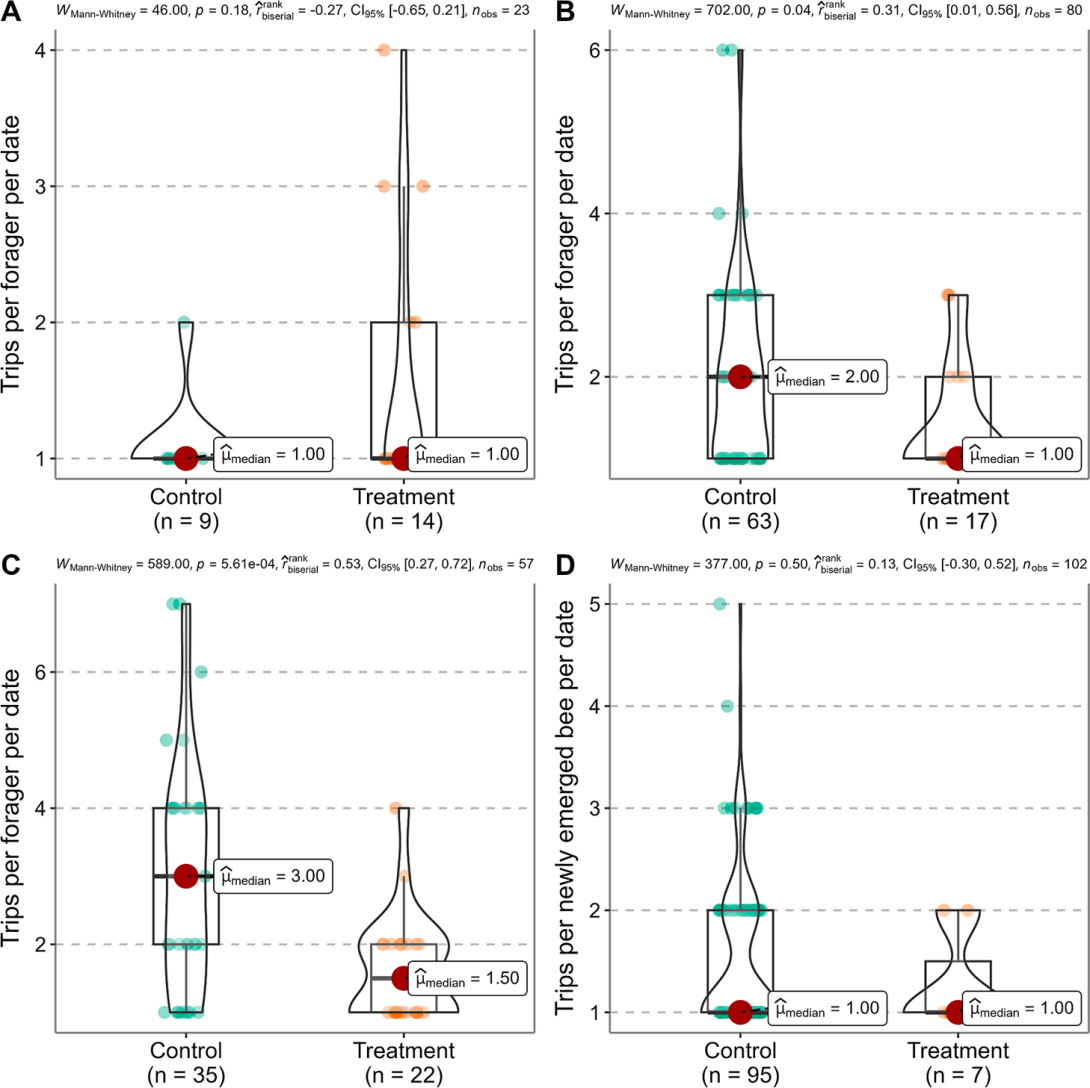
Comparison of the number of trips that a marked forager or newly emerged bee returning with pollen undertook per date between Control and Treatment by the Mann–Whitney *U* test (not significantly different at *P* = 0.05). (A) Marked foragers during the preexposure period (21 − 27 July 2023). (B) Marked foragers during the exposure period (28 July 2023 − 07 August 2023). (C) Marked foragers during the postexposure period (08 − 25 August 2023). (D) Marked newly emerged bees during the postexposure period (08 − 25 August 2023). Each point refers to the number of trips made by each marked forager or newly emerged bee returning with pollen per hive per date in the Control or Treatment hives.

The number of pollen foraging trips performed by marked foragers per date in Treatment did not significantly differ from that in Control before the exposure (Figure 5A). However, during the exposure and postexposure periods, we found that marked foragers in Treatment performed fewer pollen foraging trips per date than those in Control (Figure 5B,C). As for the number of pollen foraging trips performed by marked newly emerged bees per date, there was no significant difference between Control and Treatment during the postexposure period (Figure 5D). No observations were recorded for marked newly emerged bees engaged in pollen foraging in Treatment during the preexposure and exposure periods, while zero and three bees were recorded in Control during the preexposure and exposure periods, respectively. Thus, statistical analysis could not be conducted.

#### 3.2.3 Marked Bee Mortality

The total number of dead marked bees, including foragers and newly emerged bees, in Treatment was found to not significantly differ from that in Control on 27 July 2023, before the exposure (Control: 6 vs Treatment: 5; *P* = 1) and during the exposure period (Control: 12 vs Treatment: 14; *P* = 0.85). It should be noted that during the postexposure period, no marked bees (i.e., foragers or newly emerged bees) died in Control, while only one dead marked bee was observed in Treatment. As a result, the exact binomial test could not be applied to compare the two groups during this period due to zero counts.

#### 3.2.4 Recruitment of Newly Emerged Bees for Pollen Foraging

**Figure 6:**
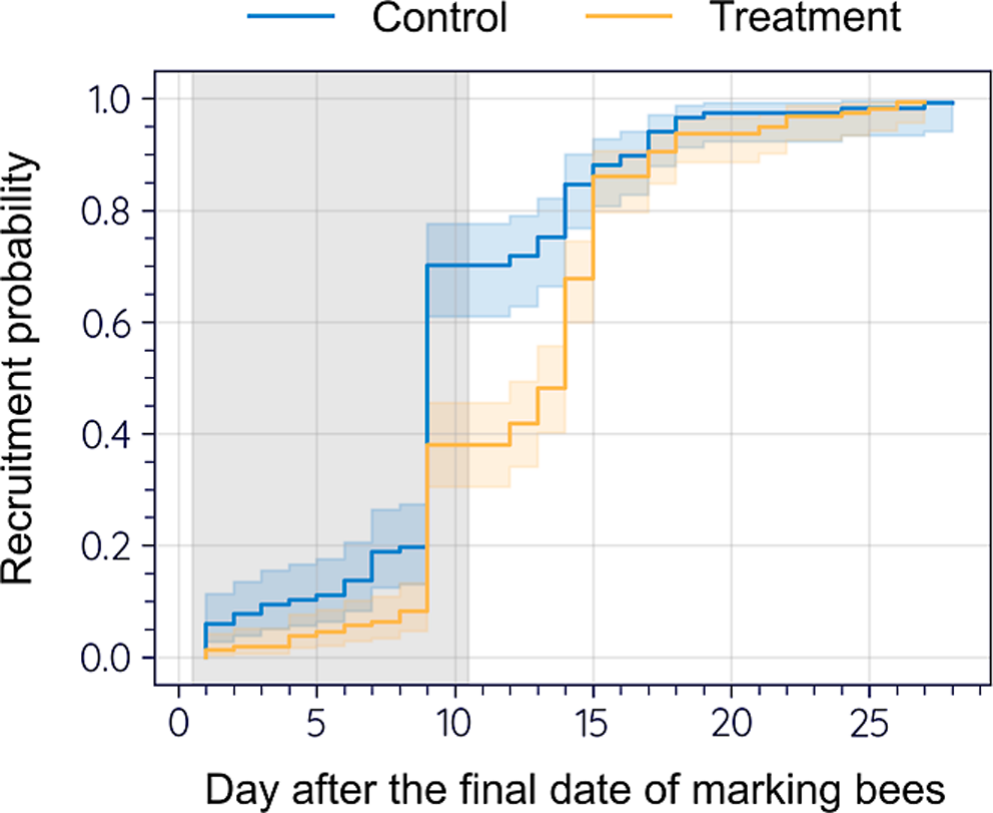
Comparison of the recruitment of marked newly emerged bees for pollen foraging between Control and Treatment by the Kaplan–Meier method and the log-rank test (not significantly different at *P* = 0.05). The median recruitment for Control and Treatment was 9 days and 14 days, respectively, from the final date of marking bees (i.e., Day 0: 27 July 2023). Statistical comparison using the log-rank test showed a significant difference in recruitment distributions between Control and Treatment (χ^2^ = 24.4, d.f. = 1, *P* = 7.839 × 10^–7^). The line represents the Kaplan–Meier estimate with the 95% confidence interval in shade. The gray area indicates the exposure period (28 July 2023 − 07 August 2023).

The recruitment of marked newly emerged bees for pollen foraging was observed in both Control and Treatment between 27 July 2023, and 25 August 2023. Recruitment was defined as the time from emergence to the first observed successful pollen foraging activity. This process was significantly delayed in Treatment compared to Control, with a median delay of 5 days (Figure 6).

#### 3.2.5 Occurrences of Drifting

**Figure 7:**
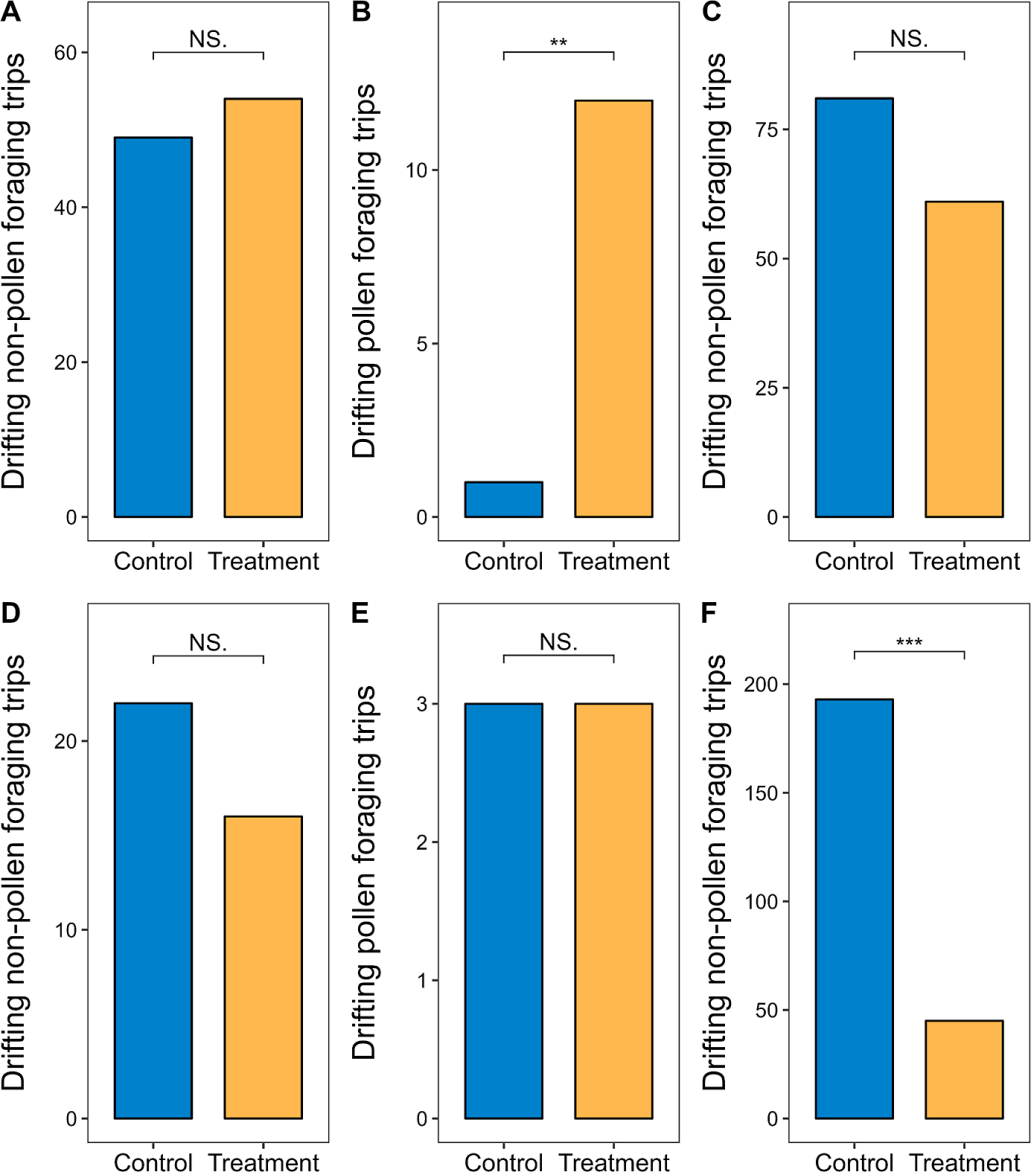
Comparison of the total number of drifting pollen or nonpollen foraging trips performed by Control and Treatment marked foragers or newly emerged bees during the exposure and postexposure periods (28 July 2023 − 07 August 2023 and 08 − 25 August 2023) by the exact binomial test (not significantly different at *P* = 0.05). (A) Marked foragers during the exposure period (Control: 49 vs Treatment: 54; *P* = 0.69). (B) Marked foragers during the exposure period (Control: 1 vs Treatment: 12; *P* = 0.003). (C) Marked newly emerged bees during the exposure period (Control: 81 vs Treatment: 61; *P* = 0.11). (D) Marked foragers during the postexposure period (Control: 22 vs Treatment: 16; *P* = 0.42). (E) Marked foragers during the postexposure period (Control: 3 vs Treatment: 3; *P* = 1). (F) Marked newly emerged bees during the postexposure period (Control: 193 vs Treatment: 45; *P* < 2.2 × 10^–16^). NS. indicates not significantly different at *P* = 0.05. ** Significance at *P* < 0.01. *** Significance at *P* < 0.001.

Marked honeybees could forage for both nectar and pollen during their lifetime, as it is uncommon for honeybees to exclusively collect pollen.^43^ Therefore, the total number of drifting pollen and nonpollen foraging trips performed by marked foragers and newly emerged bees was counted to assess differences between bees that never entered the treated hives (Control) and those that did (Treatment) during the exposure and postexposure periods (28 July 2023 − 07 August 2023 and 08 − 25 August 2023, respectively) (Figure 7).

During the exposure period, Treatment marked foragers made a significantly higher total number of drifting pollen foraging trips than Control marked foragers (Figure 7B). However, there was no significant difference in the total number of drifting nonpollen foraging trips between Treatment and Control marked foragers or newly emerged bees (Figure 7A,C). No drifting pollen foraging trips were observed for Control or Treatment marked newly emerged bees during this period, which made statistical analysis infeasible.

During the postexposure period, the total number of drifting nonpollen foraging trips by Treatment marked foragers did not differ significantly from that by Control marked foragers (Figure 7D). Both groups also performed the same total number of drifting pollen foraging trips with no significant difference observed (Figure 7E). In contrast, Control marked newly emerged bees made a significantly higher total number of drifting nonpollen foraging trips than Treatment marked newly emerged bees (Figure 7F), due to the delayed foraging initiation in Treatment bees (Figure 6). No drifting pollen foraging trips were observed for newly emerged bees in either group during the postexposure period, which prevented statistical analysis.

## 4 Discussion

In this study, we were able to reproduce the colony-level results observed in the 2019 feeding experiments. These two studies, conducted in different years and during different colony development stages (spring for the 2019 experiments and late summer for the 2023 experiments), had consistent outcomes. Given the high variability in behaviors and traits of honeybee colonies, these results suggest that the design of our feeding experiments is robust for investigating flight activities and behaviors when bees are under controlled pesticide exposure within the hive. As a result, this design has the potential to serve as a new protocol for ecological risk assessments of pesticides, in particular in evaluating honeybee flight activities and behaviors.

Our study confirmed that sublethal doses of neonicotinoids do not significantly increase forager mortality,^27^ but do affect foraging behaviors, consistent with previous findings.^11,14,17,18,22^ At the colony level, the reduced number of returning pollen foragers was directly associated with neonicotinoid exposure (Figure 2), as both Control and Treatment hives had similar overall colony conditions before exposure and were located at the same site, facing identical environmental conditions (e.g., resource availability in the surrounding landscape, weather, etc.) throughout the study period. Therefore, our observations are robust and reliable. We provided the first evidence that these doses selectively reduce pollen foraging with minimal to no effect on nectar foraging (Figure 2 and Wang et al.^23^). This differentiation between pollen and nectar foraging is particularly significant, as pollen is critical for brood development, and any reduction in pollen collection could have long-term consequences for colony health and sustainability.^44−46^ As demonstrated in our study, the decline in pollen due to inhibited pollen foraging at the colony level caused by the exposure (Figure S4) could lead to a significant decrease in brood production during the postexposure period (Figure S3). In contrast, nectar availability was significantly higher in Treatment than in Control during this period (Figure S5), likely due to the significant loss of brood (Figure S3).

At the individual level, the model predictions derived from the BEEHAVE simulations, i.e., given a limited number of hours with favorable weather conditions on any given day, an increase in the duration of each pollen foraging trip will reduce the total number of pollen foraging trips a forager can complete daily,^23^ were now confirmed by our 2023 Reidentification experiments. The experiments showed that sublethal doses of imidacloprid significantly prolonged the duration of pollen foraging trips, which in turn reduced the number of pollen foraging trips that a forager could undertake per day (Figures 4 and 5). Similar changes in foraging behaviors were also observed, for example, by Schneider et al.^11^ and Shi et al.,^13^ despite focusing on nectar foraging in controlled experimental settings where bees were directly fed or contaminated with imidacloprid. In our study, however, this decrease in pollen foraging efficiency led to a decline in overall colony-level pollen foraging (Figure 2A), which effectively reduced the total amount of pollen collected by the colony (Figure S4).

Such a reduction in pollen foraging efficiency at the individual level can have cascading effects on the colony, which results in decreased brood production (Figure S3) due to insufficient pollen availability (Figure S4). This reduction in pollen availability, caused by decreased overall colony-level pollen foraging (Figures 2A and 3A), can potentially weaken colony resilience.^44^ Furthermore, reduced brood production (Figure S3) can, in turn, lower resource demand at the colony level, which further inhibits pollen foraging (Figure 3A), as indicated by shorter pollen foraging trip durations (Figure 4C) and fewer pollen foraging trips per date (Figure 5C), occurred during the postexposure period. This aligns with general agreement that pollen foraging is demand-driven.^47,48^ In addition, reduced pollen foraging can be exacerbated by increased occurrences of drifting pollen foraging trips (Figure 7B) and delayed recruitment of newly emerged bees for pollen foraging (Figure 6), due to the loss and shortage of the colony’s workforce.

This reduction in pollen foraging is likely due to impaired cognitive function and altered metabolism in foragers. Our study showed that imidacloprid causes foragers to spend more time completing their pollen foraging trips (Figure 4) and increases the occurrence of drifting pollen foraging trips, where foragers inadvertently return to colonies other than their own (Figure 7B). These behaviors indicate a breakdown in spatial memory and navigational skills, which may further support the finding that neuroactive pesticides impair cognitive functions in honeybees.^49,50^ Furthermore, research has demonstrated that pesticides can affect honeybee metabolism.^16,51,52^ It appears that pollen foraging consumes more energy than nectar foraging.^53^ As a result of altered metabolism, pesticide-exposed pollen foragers may require more time to complete foraging trips and to rest and recover in the hive upon their return. Similar resting behaviors have been observed by Shi et al.^13^ and Wu et al.,^54^ who reported that worker bees exhibited excessive “day-off” behaviors under exposure to pesticides, which led to decreased foraging activities. In addition, the sublethal effects of neonicotinoids may also impair the cognitive and physical abilities required for foraging initiation in newly emerged bees, which can result in significantly delayed recruitment for pollen foraging (Figure 6). More importantly, pesticide exposure may have not only direct toxic effects on honeybees but also indirect consequences by limiting resource quantity and quality, such as pollen availability (Figure S4), leading to poor nutrition. This could further weaken their tolerance to pesticides^55,56^ and may even synergistically reduce survival when combined with pesticide exposure.^57^

We found that the neonicotinoid imidacloprid affected only pollen foraging, while it had no impact on nectar foraging. In contrast, some previous studies have shown that nectar foraging was also influenced by imidacloprid, although those studies were conducted in controlled experimental settings and bees were directly fed or contaminated with the substance (e.g., Schneider et al.,^11^ Fischer et al.,^17^ and Ohlinger et al.^20^). Semifield studies and studies with trained bees in relatively controlled experimental settings might not fully reflect the complex ecological interactions that bees experience in the real world. However, field studies taking the whole colony into account in real landscape conditions with free-flying bees can best reflect the reality, thereby providing more realistic and ecologically relevant results. Our study was performed in real landscape conditions with free-flying bees. This may explain the difference in results between our study and those of previous studies.

One key advantage of the state-of-the-art AI-based monitoring technology that we used is that it enables reliable observations in real landscape conditions. These reliable observations can then help identify plausible mechanisms driving colony and foraging dynamics in computer simulations, which, in turn, inform or guide empirical studies to test model predictions. This “from *in vivo* to *in silico* and back” approach can effectively disentangle mechanisms underlying observed phenomena. In particular, when using well-validated simulation models, this can also facilitate the exploration of answers to unknowns, which potentially lead to high-impact outcomes that refocus research within the field.^58^ For instance, our observation of reduced pollen foraging at the colony level, driven by prolonged foraging durations at the individual level, may play a significant role in colony collapse. Studies focusing on the direct effects of pesticides on individual honeybees in controlled environments do not necessarily allow for such conclusions.

Our AI-based monitoring technology is not capable of distinguishing between nectar and pollen foragers when they leave the hive, but it can identify returning bees with or without pollen pellets. Therefore, at the individual level, we could not precisely determine attributes such as the trip durations and the number of trips for each marked bee without pollen pellets. This is because we could not confirm whether these returning bees without pollen pellets had engaged in nectar foraging or simply left the hive for other reasons, such as ventilation, defecation, or water import.

In summary, we present a robust feeding design for investigating the effects of pesticides on honeybee colonies, as well as their flight activities and foraging behaviors in real landscape settings, which led to reliable results, in particular when sample sizes were small. This feeding design, therefore, has the potential to serve as a new protocol for ecological risk assessments of pesticides, especially concerning flight activities and behaviors in honeybees. Our study also demonstrates that BEEHAVE performs well in reproducing field observations and can then effectively guide or inform empirical studies. Combining experimental studies with computer simulations allowed us to gain insights into the mechanisms by which sublethal effects observed in individual foragers propagate to the colony level, thereby impacting overall foraging performance, resource availability, and colony dynamics. To the best of our knowledge, the current work is the first to link effects observed at the individual level to those at the colony level in honeybees under pesticide exposure, which has implications for future pesticide risk assessment.

## Supporting Information Available

Appendix A: Supplementary material, including (1) Details of the marking-and-tracking method, (2) Fig. S1. Layout and placement of hives in the current study, (3) Table S1. Comparison of the number of dead adult workers between Control and Treatment during the study period by the Welch’s *t*-test, (4) Fig. S2. Comparison of the number of adult worker bees between Control and Treatment during the preexposure, exposure, and post-exposure periods by the Welch’s *t*-test, (5) Fig. S3. Comparison of the number of brood cells between Control and Treatment during the preexposure, exposure, and post-exposure periods by the Welch’s *t*-test, (6) Fig. S4. Comparison of the number of pollen cells between Control and Treatment during the preexposure, exposure, and post-exposure periods by the Welch’s *t*-test and the Mann−Whitney *U* test, (7) Fig. S5. Comparison of the number of nectar cells between Control and Treatment during the preexposure, exposure, and post-exposure periods by the Welch’s *t*-test, and (8) Fig. S6. Comparison of sugar solution consumption between Control and Treatment during the exposure period by the Mann−Whitney *U* test (PDF)
Appendix B: Source data, including information and datasets on (1) weather, (2) colony assessments, (3) adult worker mortality, (4) feeding consumption, (5) flight activities, (6) marked bees, (7) marked bee mortality, (8) pollen foraging trip durations, (9) pollen foraging trips, (10) recruitment of marked bees, (11) drifting nonpollen foraging trips, and (12) drifting pollen foraging trips (XLSX)
