# Supplementary material for: Reduced Honeybee Pollen Foraging under Neonicotinoid Exposure: Exploring Reproducible Individual and Colony Level Effects in the Field Using AI and Simulation
Source: Environ Sci Technol. 2025 Mar 7;59(10):4883–92. doi: 10.1021/acs.est.4c13656 (PMC11924214; doi:10.1021/acs.est.4c13656)
Supplement: Supplementary file 1 — es4c13656_si_001.pdf [file es4c13656_si_001.pdf]

Supplementary material:

Reduced honeybee pollen foraging under neonicotinoid exposure: exploring reproducible individual and colony level effects in the field using AI and simulation

Ming Wang[1,*], Frederic Tausch[2], Katharina Schmidt[2], Matthias Diehl[2,3], Silvio Knaebe[4], Holger Bargen[4], Farnaz Faramarzi[4], Volker Grimm[1,5,*]

[1] Helmholtz Centre for Environmental Research – UFZ, Department of Ecological Modelling, Permoserstr. 15, 04318, Leipzig, Germany

[2] apic.ai GmbH, Melanchthonstraße 2, 76131 Karlsruhe, Germany

[3] FZI Research Center for Information Technology, Haid-und-Neu-Str. 10-14, 76131, Karlsruhe, Germany

[4] Eurofins Agroscience Services Ecotox GmbH, Eutinger Str. 24, 75223, Niefern-Öschelbronn, Germany

[5] University of Potsdam, Department of Plant Ecology and Nature Conservation, Zeppelinstraße 48 A, 14471, Potsdam-Golm, Germany

* Corresponding author: ming.wang@ufz.de; volker.grimm@ufz.de

Supplementary material includes: 9 pages, 6 figures and 1 table

Contents

Details of the marking-and-tracking method S2

Fig. S1. Layout and placement of hives in the current study. S3

Table S1. Comparison of the number of dead adult workers between Control and Treatment during the study period by the Welch's *t*-test. S4

Fig. S2. Comparison of the number of adult worker bees between Control and Treatment during the pre-exposure, exposure, and post-exposure periods by the Welch's *t*-test. S5

Fig. S3. Comparison of the number of brood cells between Control and Treatment during the pre-exposure, exposure, and post-exposure periods by the Welch's *t*-test. S6

Fig. S4. Comparison of the number of pollen cells between Control and Treatment during the pre-exposure, exposure, and post-exposure periods by the Welch's *t*-test and the Mann−Whitney *U* test. S7

Fig. S5. Comparison of the number of nectar cells between Control and Treatment during the pre-exposure, exposure, and post-exposure periods by the Welch's *t*-test. S8

Fig. S6. Comparison of sugar solution consumption between Control and Treatment during the exposure period by the Mann−Whitney *U* test. S9

Details of the marking-and-tracking method

Each bee was marked with a unique identifier using custom-printed opalith plates that the apic.ai camera system could detect. The system captured images of marked bees as they entered and exited the hive. The re-identification process relied on an algorithm with a detection accuracy of 99.87%, validated on a dataset of 1,511 bees (750 marked and 761 unmarked). After initial detection, manual assessments were performed to identify the specific marker ID on each bee. These manual assessments were conducted by trained personnel. While there was no quantified accuracy rate for the manual annotation process, the availability of images for each observation allowed for verification of the results.

For each marked bee, observations were collected. Data available for each observation included a timestamp, an image of the marked bee, the visual marker ID, and an indication of whether the bee carried pollen. These data facilitated key analyses, including:

- The first time a bee was observed leaving the hive (indicating recruitment to foraging).
- The last time a bee was observed at a hive (indicating potential survival).
- Each instance of the bee leaving the hive without pollen and entering with pollen (indicating pollen foraging flights).
- The duration of these pollen foraging trips (assessing foraging efficiency and potential cognitive impairments).

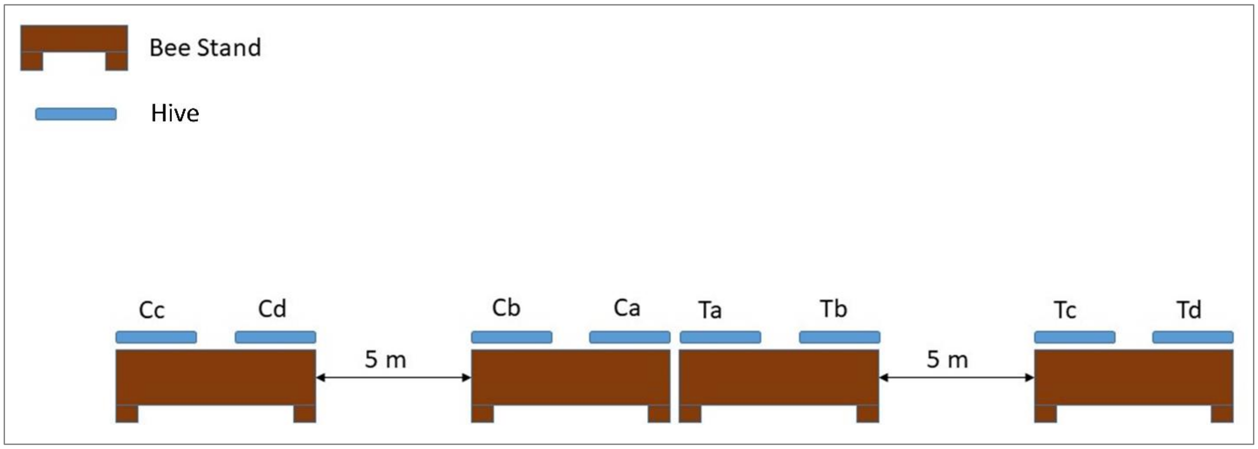

Fig. S1. Layout and placement of hives in the current study.

Table S1. Comparison of the number of dead adult workers between Control and Treatment during the study period by the Welch's *t*-test.

Period	Date	Control (mean ± SD; n = 4)	Treatment (mean ± SD; n = 4)	*t*	*d.f.*	*P*
Pre-exposure	21-July-2023	3.25 ± 3.4	6 ± 4.4	-0.99	5.65	0.36
	22-July-2023	10.5 ± 5.32	13.75 ± 5.56	-0.84	5.99	0.43
	23-July-2023	20.75 ± 8.22	31.75 ± 17.1	-1.16	4.32	0.31
	24-July-2023	19 ± 14	21.75 ± 7.93	-0.34	4.75	0.75
	25-July-2023	24 ± 8.12	25.25 ± 6.85	-0.24	5.83	0.82
	26-July-2023	19.25 ± 18	30 ± 10.9	-1.02	4.93	0.35
	27-July-2023	38 ± 43.2	32.75 ± 16.8	0.23	3.89	0.83
Exposure	28-July-2023	10.75 ± 3.59	8.25 ± 5.8	0.73	5.01	0.50
	29-July-2023	11.5 ± 1.29	14.75 ± 6.5	-0.98	3.24	0.39
	30-July-2023	11.25 ± 5.62	18.75 ± 5.74	-1.87	6.00	0.11
	31-July-2023	7 ± 2.16	17.25 ± 11.6	-1.74	3.21	0.17
	01-August-2023	6 ± 3.46	20.75 ± 9.32	-2.97	3.81	0.04
	02-August-2023	13.25 ± 5.91	10.25 ± 3.2	0.89	4.62	0.42
	03-August-2023	11 ± 2	11.5 ± 6.14	-0.15	3.63	0.89
	04-August-2023	14.5 ± 4.65	17.75 ± 6.29	-0.83	5.53	0.44
	05-August-2023	9.25 ± 2.75	18 ± 9.31	-1.80	3.52	0.16
	06-August-2023	16.5 ± 8.19	20 ± 8.21	-0.60	6.00	0.57
	07-August-2023	16.25 ± 4.79	18.5 ± 4.8	-0.66	6.00	0.53
Post-exposure	08-August-2023	11.75 ± 2.75	14.75 ± 10	-0.58	3.45	0.60
	09-August-2023	10 ± 3.16	11 ± 9.13	-0.21	3.71	0.85
	10-August-2023	13.75 ± 6.9	8.5 ± 5.45	1.19	5.69	0.28
	11-August-2023	18 ± 11	21.75 ± 19.6	-0.33	4.71	0.75
	12-August-2023	33.25 ± 7.85	23.5 ± 10.2	1.51	5.63	0.18
	13-August-2023	23.25 ± 14.5	17.25 ± 4.79	0.79	3.65	0.48
	14-August-2023	14.25 ± 4.57	13 ± 6.68	0.31	5.30	0.77
	15-August-2023	14.5 ± 4.65	19.75 ± 10.3	-0.93	4.17	0.40
	16-August-2023	13 ± 3.83	13 ± 8.08	0.00	4.28	1.00
	17-August-2023	22.25 ± 8.54	30.75 ± 12.2	-1.14	5.36	0.30
	18-August-2023	14.75 ± 3.77	20 ± 10.3	-0.96	3.79	0.40
	19-August-2023	14.75 ± 7.93	10 ± 6.38	0.93	5.74	0.39
	20-August-2023	13 ± 3.74	13.5 ± 8.7	-0.11	4.07	0.92
	21-August-2023	10 ± 4.97	17.25 ± 16.2	-0.86	3.56	0.45
	22-August-2023	23.75 ± 13.7	11.5 ± 3.42	1.73	3.37	0.17
	23-August-2023	15.75 ± 4.79	14.75 ± 10.9	0.17	4.11	0.87
	24-August-2023	17 ± 5.1	18.75 ± 10.9	-0.29	4.25	0.78
	25-August-2023	37.25 ± 16	23.5 ± 10.5	1.44	5.18	0.21

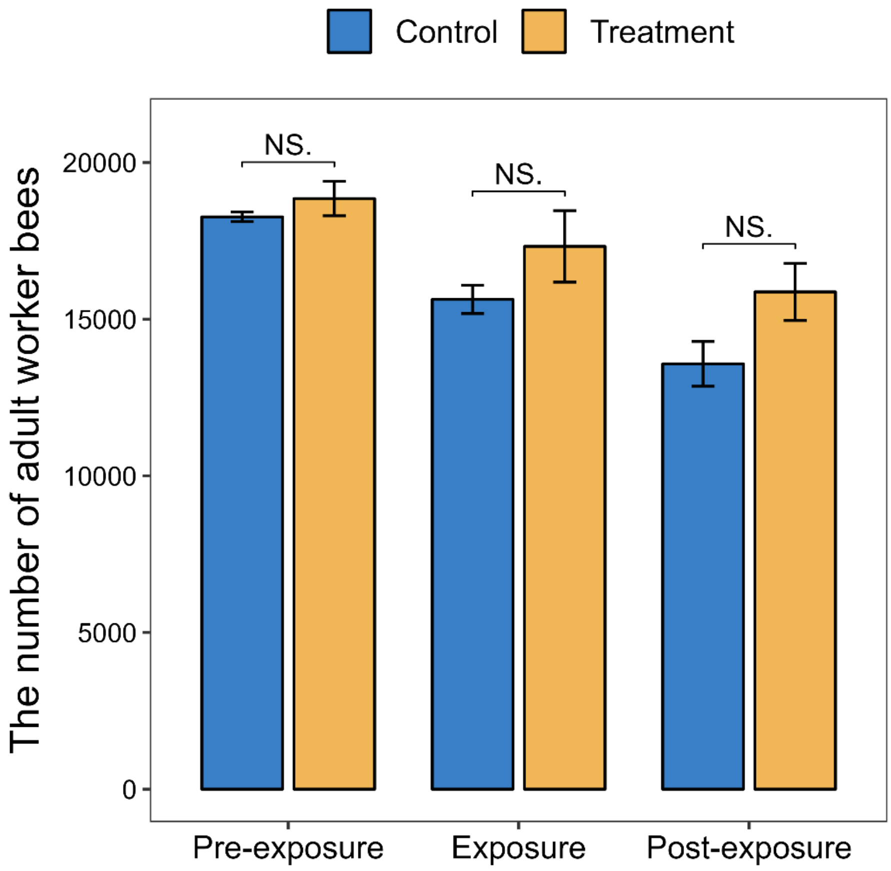

Fig. S2. Comparison of the number of adult worker bees between Control and Treatment during the pre-exposure, exposure, and post-exposure periods by the Welch's *t*-test. Control (mean ± SE: 18265 ± 152; n = 4) vs. Treatment (mean ± SE: 18850 ± 550; n = 4) during the pre-exposure period (t = -1.03, *d.f.* = 3.46, P = 0.37). Control (mean ± SE: 15632 ± 454; n = 4) vs. Treatment (mean ± SE: 17322 ± 1141; n = 4) during the exposure period (t = -1.38, *d.f.* = 3.93, P = 0.24). Control (mean ± SE: 13574 ± 713; n = 12) vs. Treatment (mean ± SE: 15871 ± 914; n = 12) during the post-exposure period (t = -1.98, *d.f.* = 20.8, P = 0.06). NS. indicates not significantly different at P = 0.05.

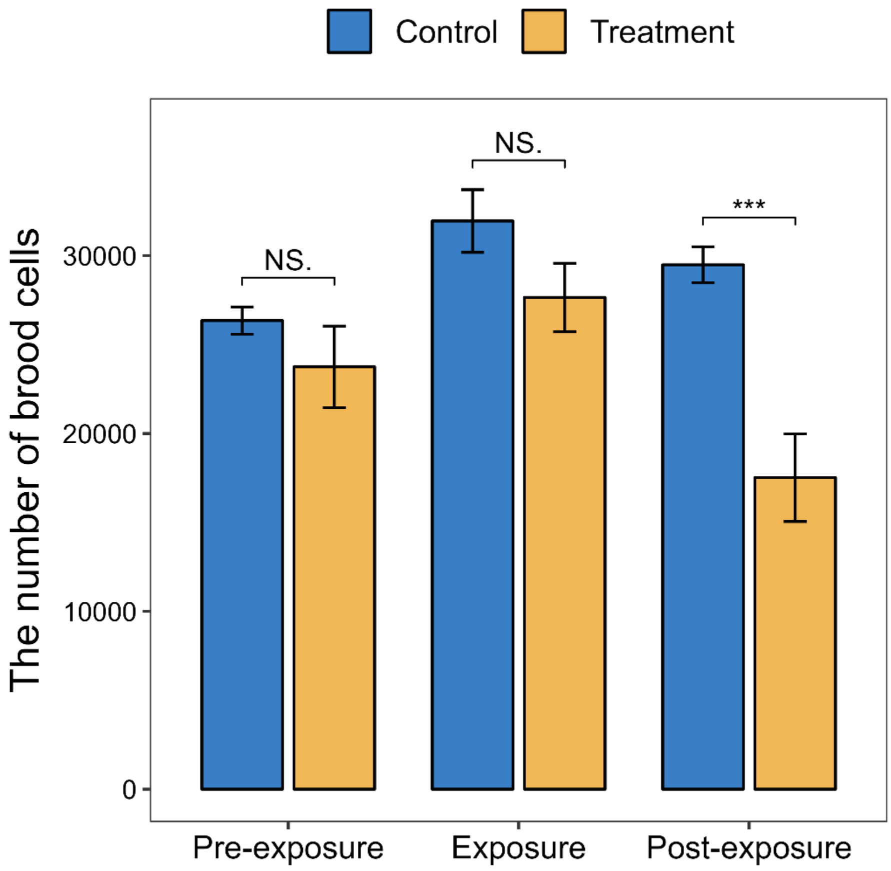

Fig. S3. Comparison of the number of brood cells between Control and Treatment during the pre-exposure, exposure, and post-exposure periods by the Welch's *t*-test. Control (mean ± SE: 26350 ± 768; n = 4) vs. Treatment (mean ± SE: 23750 ± 2290; n = 4) during the pre-exposure period (t = 1.08, *d.f.* = 3.67, P = 0.35). Control (mean ± SE: 31950 ± 1763; n = 4) vs. Treatment (mean ± SE: 27650 ± 1924; n = 4) during the exposure period (t = 1.65, *d.f.* = 5.95, P = 0.15). Control (mean ± SE: 29486 ± 1013; n = 12) vs. Treatment (mean ± SE: 17517 ± 2465; n = 12) during the post-exposure period (t = 4.49, *d.f.* = 14.6, P = 0.00046). NS. indicates not significantly different at P = 0.05. ******* Significance at $P < 0.001$.

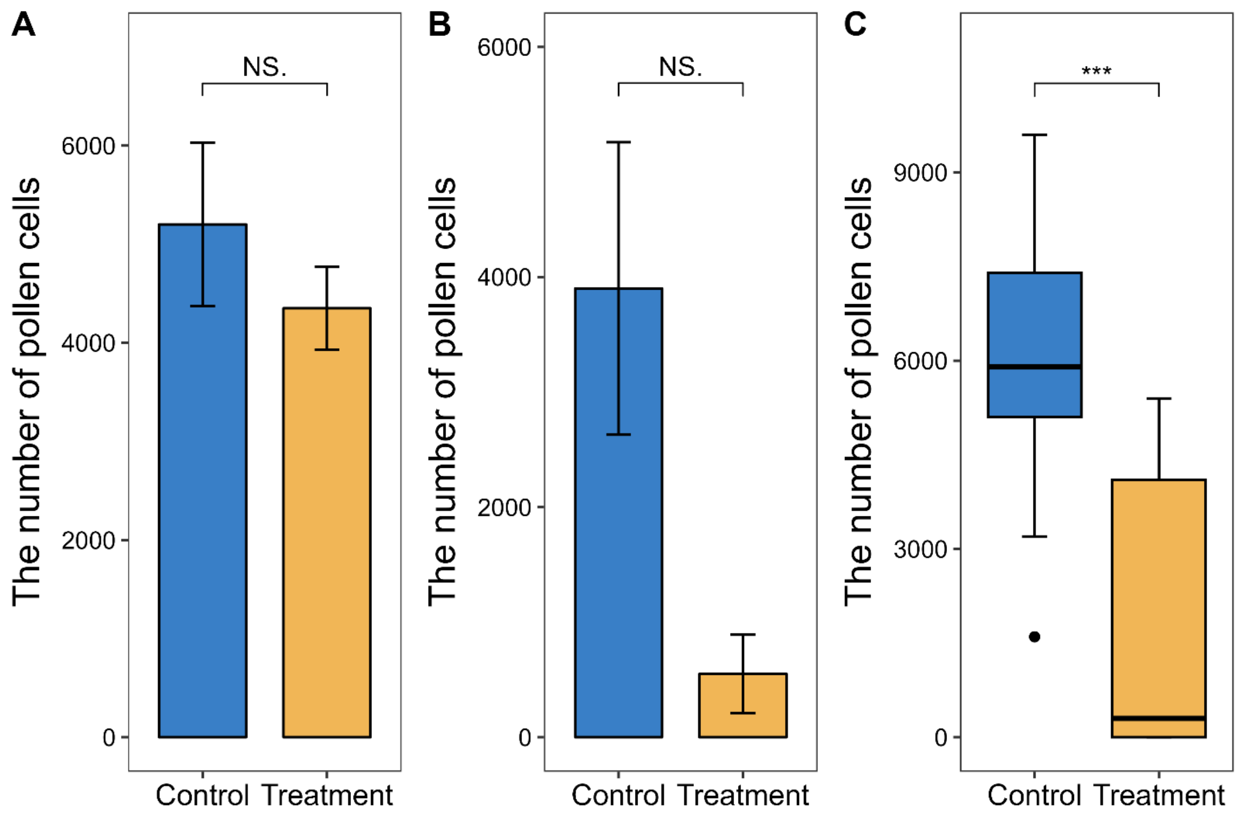

Fig. S4. Comparison of the number of pollen cells between Control and Treatment during the pre-exposure, exposure, and post-exposure periods by the Welch's *t*-test and the Mann−Whitney *U* test. (A) Control (mean ± SE: 5200 ± 829; *n* = 4) vs. Treatment (mean ± SE: 4350 ± 419; *n* = 4) during the pre-exposure period (*t* = 0.92, *d.f.* = 4.44, *P* = 0.4). (B) Control (mean ± SE: 3900 ± 1271; n = *4*) vs. Treatment (mean ± SE: 550 ± 340; *n* = 4) during the exposure period (*t* = 2.55, *d.f.* = 3.43, *P* = 0.07). (C) Control [median (IQR): 5900 (5100 – 7400); *n* = 12] vs. Treatment [median (IQR): 300 (0 – 4100); *n* = 12] during the post-exposure period (*W* = 133, *P* = 0.00046). NS. indicates not significantly different at *P* = 0.05. ******* Significance at *P* < 0.001. • represents outliers.

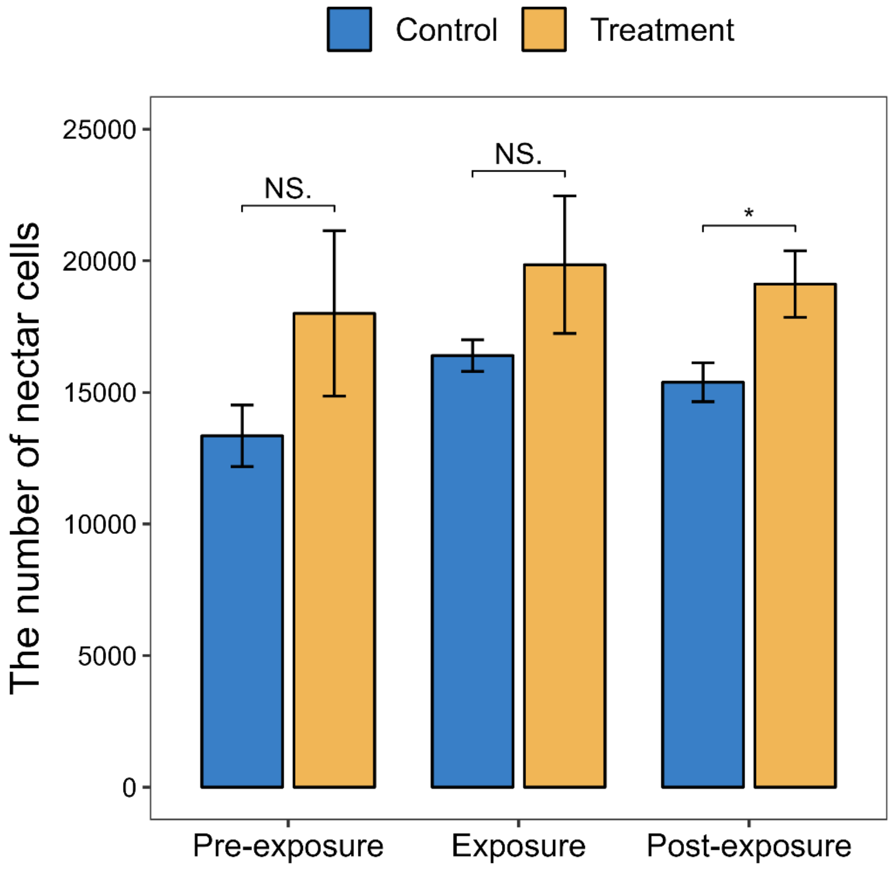

Fig. S5. Comparison of the number of nectar cells between Control and Treatment during the pre-exposure, exposure, and post-exposure periods by the Welch's *t*-test. Control (mean ± SE: 13350 ± 1167; $n = 4$) vs. Treatment (mean ± SE: 18000 ± 3144; $n = 4$) during the pre-exposure period ($t = -1.39$, $d.f. = 3.81$, $P = 0.24$). Control (mean ± SE: 16400 ± 600; $n = 4$) vs. Treatment (mean ± SE: 19850 ± 2612; $n = 4$) during the exposure period ($t = -1.29$, $d.f. = 3.32$, $P = 0.28$). Control (mean ± SE: 15383 ± 738; $n = 12$) vs. Treatment (mean ± SE: 19117 ± 1266; $n = 12$) during the post-exposure period ($t = -2.55$, $d.f. = 17.7$, $P = 0.02$). NS. indicates not significantly different at $P = 0.05$. ***** Significance at $P < 0.05$.

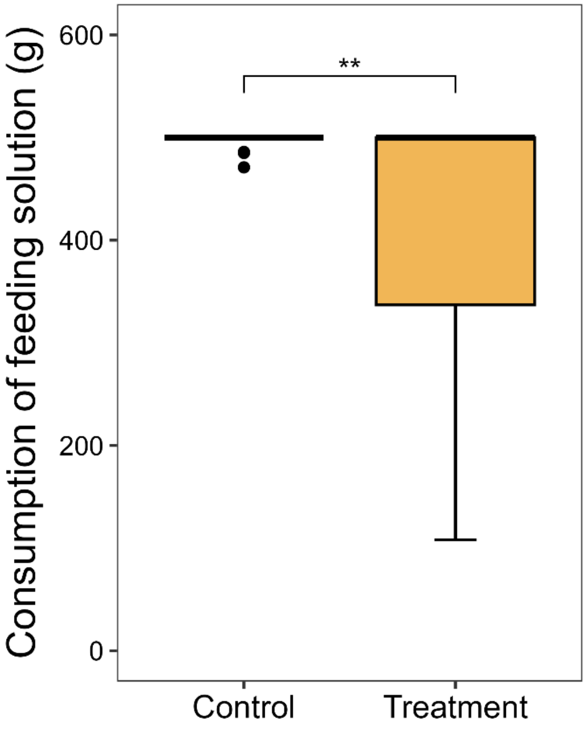

Fig. S6. Comparison of sugar solution consumption between Control and Treatment during the exposure period by the Mann−Whitney *U* test. Control [median (IQR): 500 (500 – 500); $n = 40$] vs. Treatment [median (IQR): 500 (337 – 500); $n = 40$] during the exposure period ($W = 1040$, $P = 0.00127$). ****** Significance at $P < 0.01$. • represents outliers.
